# 1143. Meningococcal Vaccination Coverage Disparities in the United States: An Analysis with 2016-2021 National Immunization Survey-Teen Data

**DOI:** 10.1093/ofid/ofad500.984

**Published:** 2023-11-27

**Authors:** Oscar Herrera-Restrepo, Marta Kwiatkowska, Nelson Ndegwa, Sara Poston, Michael Ganz

**Affiliations:** GSK, Philadelphia, Pennsylvania; Evidera, London, England, United Kingdom; Evidera, London, England, United Kingdom; GSK, Philadelphia, Pennsylvania; Evidera, Inc., Waltham, Massachusetts

## Abstract

**Background:**

The US Advisory Committee on Immunization Practices recommends meningococcal vaccination against serogroups A, C, W, and Y (MenACWY) for 11–12 year-olds (y/o) with a booster at 16; and against serogroup B (MenB) for 16–23 y/o under shared clinical decision making (SCDM). We used a large US survey to assess vaccination coverage across vaccines, geography and race/ethnicity.

**Methods:**

This was a cross-sectional analysis using 2016-2021 National Immunization Survey-Teen survey data on MenACWY (≥1 dose [d]) in 13 y/o, MenACWY (≥2 d) and MenB (≥1 d) in 17 y/o. Data were collected via phone interviews with provider confirmation. We assessed coverage throughout the calendar year for MenACWY (≥1 d) and MenB (≥1 d) after the interview date, and for MenACWY (≥2 d) at the interview date.

**Results:**

MenACWY coverage (≥1 d) in 13 y/o evolved from 82.71% (2016) to 86.42% (2021). MenACWY coverage (≥1 d) in 13-17 y/o changed from 83.36% (2016) to 89.82% (2021). MenB coverage (≥1 d) in 17 y/o changed from 5.63% (2016) to 34.05% (2021).

Geographic coverage was more variable moving from MenACWY (≥1 d) in 13 y/o (74.44% Pacific region 2016 to 91.00% New England region 2021) to MenACWY (≥2 d) in 17 y/o (21.05% East South Central region 2016 to 75.68% Mid-Atlantic region 2021) and MenB (≥1 d) in 17 y/o (2.34% Pacific region 2016 to 45.29% South Atlantic region 2021). MenACWY coverage (≥1 d) in 13 y/o was consistent across race/ethnicity, while 17 y/o Hispanic had lower MenACWY coverage (≥2 d) in 2021 (50.63%). For MenB (≥1 d) in 17 y/o, coverage varied by race/ethnicity; coverage in Hispanic and Non-Hispanic (NH) Black populations increased from 5.55% and 7.55% to 33.58% and 42.33% over 5 years, respectively. In NH White population, coverage yearly increased from 5.29% to 33.88%, while it only progressed from 5.34% to 27.17% in other NH + Multiple race population.

**Conclusion:**

Meningococcal vaccination disparities persist across vaccines, ages, geography, and race/ethnicity (MenB only). Disparities increase with age, and with SCDM- versus routinely- recommended vaccines (MenB vs. MenACWY). Top performing states show that better coverage is possible (∼70% MenB coverage in North Dakota vs. 34% nationally in 2021), pointing at the need to reduce access barriers to vaccination, e.g., via simplified vaccine recommendations/schedules.

**Disclosures:**

**Oscar Herrera-Restrepo, PhD**, GSK: Stocks/Bonds **Sara Poston, PharmD**, GSK: Stocks/Bonds

